# Moralizing partisanship when surrounded by copartisans versus in mixed company

**DOI:** 10.1093/pnasnexus/pgaf105

**Published:** 2025-04-01

**Authors:** Michalis Mamakos, Tessa E S Charlesworth, Eli J Finkel

**Affiliations:** Kellogg School of Management, Northwestern University, 2211 Campus Drive, Evanston, IL 60208, USA; Kellogg School of Management, Northwestern University, 2211 Campus Drive, Evanston, IL 60208, USA; Kellogg School of Management, Northwestern University, 2211 Campus Drive, Evanston, IL 60208, USA; Department of Psychology, Northwestern University, 2029 Sheridan Road, Evanston, IL 60208, USA

**Keywords:** partisan asymmetries, morality, social media, word embeddings, computational social science

## Abstract

Partisans tend to view their ingroup as moral and their outgroup as immoral. Here, we examine whether left-wing (LW) and right-wing (RW) Reddit users (N>1,000,000) express these partisan moralization views. Critically, we compare the rates of partisan moralization not only when users are in contexts (subreddits) of their ingroup (e.g. r/democrats, r/vegetarian, r/Conservative, r/Hunting) but also when in mixed-company contexts populated mostly by users without partisan engagement (e.g. r/Music, r/Parenting). First, we developed four word embedding models—two for the users of each political side, one based on their comments in their ingroup contexts and one based on their comments in mixed-company contexts. Then, we evaluated the words of each model on two semantic dimensions, partisanship and morality, and we examined their correlation as an indicator of the expressed partisan moralization. Our first analysis demonstrated that LW users express moralized partisanship to a similar degree when surrounded by copartisans and when in mixed company. However, the moralized partisanship expressed by RW users in mixed company is weaker than that they express among copartisans, as well as that expressed by LW users in mixed company. In a second analysis, we divided partisan contexts based on whether they are inherently political (e.g. r/democrats) or not (e.g. r/vegetarian). This second analysis revealed that RW users express moralized partisanship more strongly than LW users in inherently political contexts, but right- and left-wingers are similar in nonpolitical partisan contexts. The discussion considers potential explanations for these asymmetries.

## Introduction

Partisan moralization constitutes a core component of the surging polarization (1). People with higher moral convictions display greater partisan bias (2) and are more likely to endorse undemocratic processes to achieve their partisan goals (3). These moral convictions also result in social distancing from people with different beliefs (4), which further deteriorates intergroup relations (5). Therefore, partisan moralization is an important issue that undermines the democratic functioning and social cohesion of today’s society.

Past research has examined asymmetries in partisan moralization, with mixed results about whether it is higher among liberals (6) or conservatives (7). However, it is unknown whether the degree to which this moralization is expressed varies across social contexts. Moral convictions are resistant to peer influence (3), but there is no such knowledge about the degree of their expression. Much of the dissemination of partisan moral content today happens on social media (8), where users can join both political and nonpolitical communities (although the latter often host political discussions nonetheless (9)). Here, we ask: Do people express partisan moralization to a different degree when among their copartisans versus in mixed company?

The present report examines the comments of left-wing (LW) and right-wing (RW) Reddit users in partisan subreddits (those that are disproportionately populated by users of one side) and in mixed-company subreddits (those that are nonpolitical in content, and populated mostly by users without partisan engagement and similarly by users of each side). For each of the two groups of users, we developed two word embedding models (10), one based on their comments in their respective ingroup subreddits (e.g. r/democrats, r/vegetarian, r/Conservative, r/Hunting), and one based on their comments in mixed-company subreddits (e.g. r/Music, r/Parenting). For each of the four models separately, we constructed a semantic dimension of partisanship by projecting (11) each word onto the average of vector representation differences of pairs of words (e.g. $\vec{republican}-\vec{democrat}$) from a list we devised. We constructed a semantic dimension of morality similarly, with pairs of words (e.g. $\vec{moral}-\vec{immoral}$). Both partisanship and morality dimensions were extensively validated (SI Appendix).

## Results

**Fig. 1.**
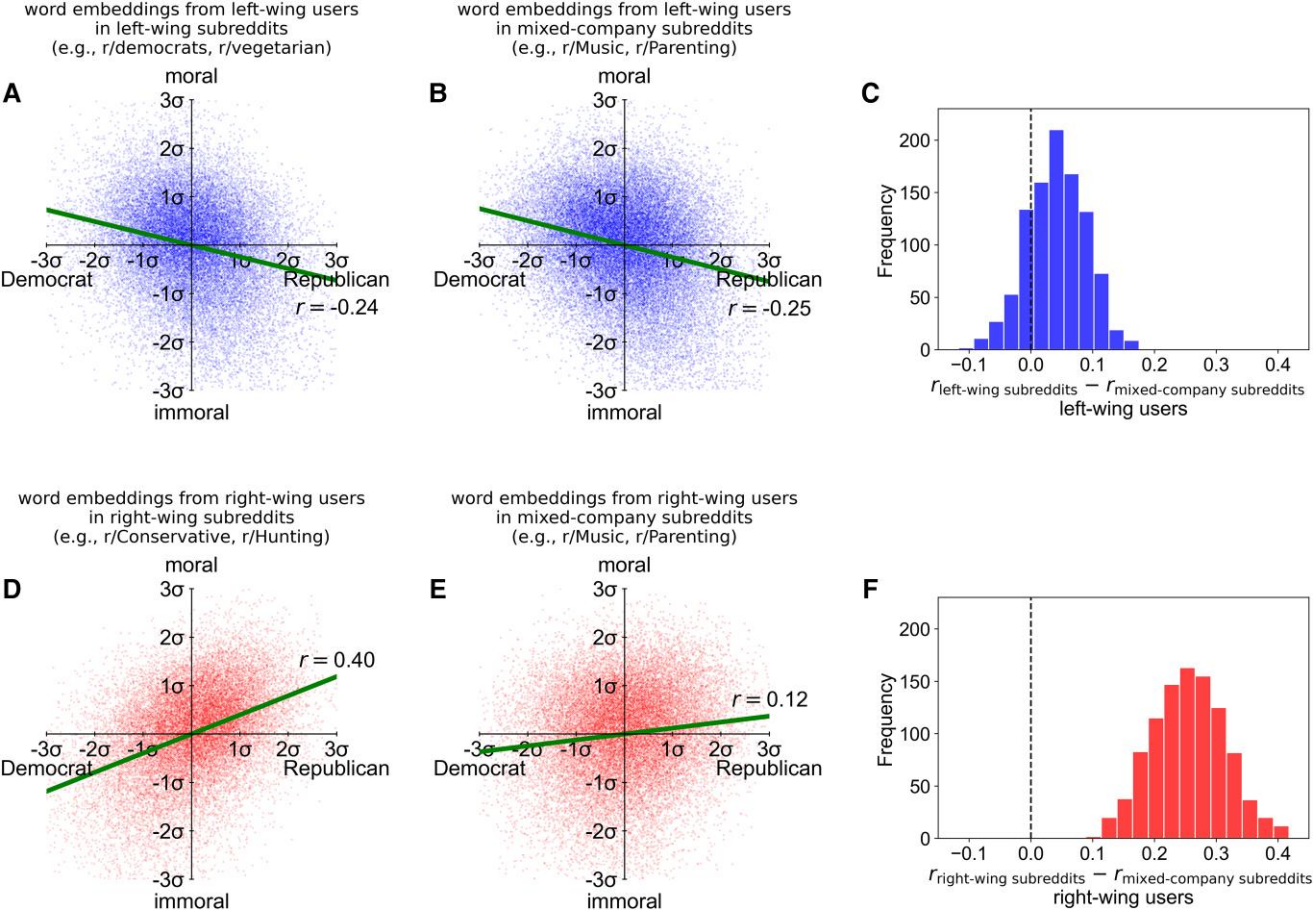
Partisanship and morality of words from the embedding models of a) LW users in LW subreddits, b) LW users in mixed-company subreddits, d) RW users in RW subreddits, and e) RW users in mixed-company subreddits; c and f) depict bootstrapped distributions of differences of correlations between partisanship and morality across partisan and mixed-company subreddits for LW users and RW users, respectively.

Figure 1a depicts the z-scores in the semantic dimensions of partisanship (*x*-axis) and morality (*y*-axis) of the words in the embedding model developed with the comments of LW users in LW subreddits. These two dimensions were correlated (r=−0.24, $P_{\mathrm{bootstrap}}<0.001$). Results in Figure 1b reveal that LW users expressed comparable moralized partisanship in mixed-company subreddits (r=−0.25, $P_{\mathrm{bootstrap}}<0.001$). A bootstrapped distribution of the difference between these correlations suggests no statistically significant difference ($P_{\mathrm{bootstrap}}>0.39$; Fig. 1c). Thus, LW users expressed moralized partisanship as strongly in mixed company as when surrounded by their ingroup left-wingers. In contrast, RW users expressed moralized partisanship more strongly in their ingroup partisan subreddits (r=0.40, $P_{\mathrm{bootstrap}}<0.001$; Fig. 1d) than in mixed-company subreddits (r=0.12, $P_{\mathrm{bootstrap}}<0.05$; Fig. 1e). The results in Figure 1f demonstrate that this difference was significant ($P_{\mathrm{bootstrap}}<0.001$). Cross-group comparisons revealed that the correlation between partisanship and morality was stronger for RW users in RW subreddits than for LW users in LW subreddits ($P_{\mathrm{bootstrap}}<0.001$), whereas this correlation was stronger for LW users than for RW users in mixed-company subreddits ($P_{\mathrm{bootstrap}}<0.02$).

Next, we examined whether the degree of partisan moralization expressed in partisan subreddits (see Fig. 1a and d) differs based on whether these subreddits are inherently political (e.g. r/democrats, r/Conservative) or not (e.g. r/vegetarian, r/Hunting). We developed four additional word embedding models (LW users in political LW subreddits, LW users in nonpolitical LW subreddits, RW users in political RW subreddits, and RW users in nonpolitical RW subreddits). Again, all semantic dimensions were validated (SI Appendix).

**Fig. 2.**
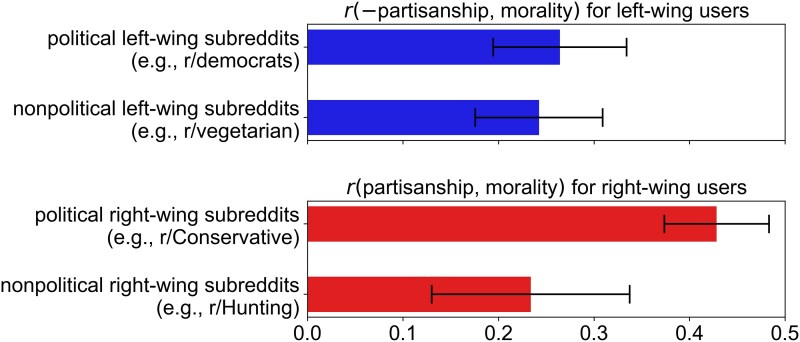
Correlations between the partisanship and the morality of words in political and nonpolitical partisan subreddits, with 95% CI.

In Figure 2, the correlation between partisanship and morality was similar for (i) LW users in political LW subreddits (r=0.26; dimension of partisanship is reversed), (ii) LW users in nonpolitical LW subreddits (r=0.24), and (iii) RW users in nonpolitical RW subreddits (r=0.23). However, the moralized partisanship expressed by RW users in political RW subreddits was significantly stronger (r=0.43, $P_{\mathrm{bootstrap}-\mathrm{Bonferroni}}<0.01$). These results show that our previous finding about the stronger partisan moralization of RW users compared to LW users in their respective ingroup contexts is driven by differences in political, rather than nonpolitical, contexts.

Our final analysis examined the words that each political side associated with the outgroup in mixed-company subreddits (which are all nonpolitical in content). The left column of Table 1 shows the top-10 words that LW users associated with RW targets in mixed company. These words centered on religion and oppression, and were, on average, immoral (M=−1.35σ, P<0.01). In contrast, the right column of Table 1 shows the top-10 words that RW users associated with LW targets in mixed company. Here, the words lacked a specific theme and were, on average, not consistently moralized (M=0.21σ, P>0.38). This qualitative analysis aligns with the earlier quantitative results showing that LW users expressed stronger partisan moralization than RW users in mixed company. It is also notable that, compared to LW users, RW users associated fewer identity-relevant words with the outgroup. Perhaps, this difference in identity-relevance means that the partisanship dimension was noisier for RW users.

**Table 1.** Top-10 words associated with the outgroup, and their morality z-scores in parentheses.

Words that left-wingers associated with right-wingers in mixed-company subreddits (e.g. r/Music, r/Parenting)	Words that right-wingers associated with left-wingers in mixed-company subreddits (e.g. r/Music, r/Parenting)
evangelical (−0.52)	arts (−0.15)
fundamentalist (−1.62)	zoo (−0.32)
mormon (0.16)	tai (0.34)
gop (−0.87)	utopia (0.06)
batshit (−1.49)	art (0.57)
anti-science (−2.09)	grav (0.62)
abusive (−3.48)	paradise (−0.07)
homophobic (−2.13)	hostel (1.02)
evangelicals (−1.24)	waterloo (1.36)
church (−0.25)	spill (−1.28)
Average morality scores (positive = moral, negative = immoral)	
−1.35	0.21

Seed words have been excluded.

## Discussion

This work examined the semantic association between partisanship and morality as revealed by the comments of LW and RW users both in their ingroup and in mixed-company subreddits. Our findings suggest that LW users express moralized partisanship to the same degree among their copartisans and in mixed company. In contrast, for RW users the audience of a discussion is an important factor in expressing partisan moralization. RW users express moralized partisanship more strongly when among their copartisans than in mixed company—where they expressed moralized partisanship more weakly than LW users. Furthermore, our results show that the moralizing tendency of RW users is particularly strong in inherently political spaces of their ingroup.

Future work could fruitfully investigate the underlying causes of the observed asymmetries in the expression of partisan moralization. While our findings do not afford causal conclusions, it is possible that right-wingers engage in self-censorship when in mixed company. This explanation aligns with recent findings that conservatives (and moderates) feel less free to speak their minds than liberals (12). This case could have consequences for the democratic functioning of social media, especially given that left-wingers express moralized partisanship to the same degree regardless of the surrounding company. Under this explanation, another question that future research can test is whether the heightened moralization by right-wingers in political contexts is a form of reactance to the censorship they impose on themselves in other public spaces.

It is also possible that conservatives are more likely to follow rules than liberals (13), whereas liberals may ignore the different norms across different social contexts. Relatedly, liberals may be more “chronic” partisans, with more salient partisan identities than conservatives. Future work can examine whether right-wingers perceive nonpolitical RW subreddits as less populated by their copartisans than they really are. Ultimately, this research sheds light on the social media contexts that might be the most hostile to democratic communication. Targeted interventions can be informed by understanding which users in which contexts are especially likely to moralize partisanship.

## Methods

Using the partisan segregation measure of Waller and Anderson (14), we classified subreddits as LW if they had segregation at least 2 SDs below the central point of 0 ($N_{\mathrm{LWsubreddits}}=291$), and as RW if they had segregation at least 2 SDs above that central point ($N_{\mathrm{RWsubreddits}}=176$). We classified subreddits as mixed-company if they met both criteria of (i) having segregation at most 0.25 SDs away from the central point of 0 and (ii) being of nonpolitical content ($N_{\mathrm{mixed}-\mathrm{companysubreddits}}=2,078$). Mixed-company subreddits are populated mostly by users without engagement in partisan subreddits (15). Subreddits were classified as of political content if they appeared in the list of political subreddits devised by Hofmann et al. (16).

We classified as LW the users with at least 10 comments in LW subreddits, 10 comments in mixed-company subreddits, and exactly 0 comments in RW subreddits ($N_{\mathrm{LWusers}}=671,979$) in 2006–2022. Similarly, we classified as RW the users with at least 10 comments in RW subreddits, 10 comments in mixed-company subreddits, and exactly 0 comments in LW subreddits ($N_{\mathrm{RWusers}}=430,148$). Moreover, 83% of all comments were made after 2016. The partisanship and morality dimensions were validated by comparing our models’ scores with words retrieved from established (and new) dictionaries of these dimensions. Further details are reported in SI Appendix.

## Supplementary Material

pgaf105_Supplementary_Data

## Data Availability

Data and code used to generate the results are available at https://osf.io/bt64v/.
