# Supplementary material for: Moralizing partisanship when surrounded by copartisans versus in mixed company
Source: PNAS Nexus. 2025 Apr 1;4(4):pgaf105. doi: 10.1093/pnasnexus/pgaf105 (PMC11983280; doi:10.1093/pnasnexus/pgaf105)
Supplement: pgaf105_Supplementary_Data [file pgaf105_supplementary_data.pdf]

Supporting Information for

Moralizing partisanship when surrounded by co-partisans versus in mixed company

Michalis Mamakos[a,1], Tessa E. S. Charlesworth[a], Eli J. Finkel[a,b]

[a] Kellogg School of Management, Northwestern University
[b] Department of Psychology, Northwestern University

[1] Corresponding author
Email: mamakos@u.northwestern.edu

Extended Methods

Developing the word embedding models. The word embedding models were trained with the Word2vec algorithm. We selected the number of dimensions of the embeddings and the exponent that determines the negative sampling distribution based on 7 intrinsic evaluation tasks of word similarity (1-7): SimVerb-3500, WordSim-353, RareWord, SimLex-999, MEN, MTurk-287, and MTurk-771. For our four corpora corresponding to the results in Figure 1 of the main text, the results from the intrinsic evaluation tasks suggested that performance increased with embedding dimensionality, but only marginally after 300 dimensions, and so we selected this number of dimensions. Regarding the exponent for the negative sampling, the same results favored the value −0.5 suggested by Caselles-Dupré et al. (8) over the value 0.75 typically employed in models trained with the Word2vec algorithm. We set the parameter for the minimum frequency of words to 500 (discarding words with a smaller frequency than this in a corpus) because we found this beneficial for the validation of the semantic dimension of partisanship, which is described below. For all the other parameters of Word2vec, we used the default values of Gensim.[1]

Constructing and validating the semantic dimension of partisanship. To construct the semantic dimension of partisanship, we devised the following list of 6 pairs of words: [republican, democrat], [republicans, democrats], [conservative, liberal], [conservatives, liberals], [conservatism, liberalism], and [right-wing, left-wing].

Initially, we had also considered the pairs [righty, lefty], [righties, lefties], [rightist, leftist], [rightists, leftists], and [repubs, dems], but at least one of the words of each pair did not meet the minimum frequency in at least one of the four corpora corresponding to the models in Figure 1 of the main text, and thus we left out of the list these five pairs. The partisanship score of a word was computed as the cosine similarity of its vector representation to the average vector difference of the pairs of words in the list, where the differences were taken after having normalized the words in the list to have unit length. That is, considering the vector *rightleft* defined as,

$$\begin{aligned}\overrightarrow{rightleft} = &\left(\overrightarrow{republican} - \overrightarrow{democrat}\right) + \left(\overrightarrow{republicans} - \overrightarrow{democrats}\right) \\ &+ \left(\overrightarrow{conservative} - \overrightarrow{liberal}\right) + \left(\overrightarrow{conservatives} - \overrightarrow{liberals}\right) \\ &+ \left(\overrightarrow{conservatism} - \overrightarrow{liberalism}\right) + \left(\overrightarrow{right-wing} - \overrightarrow{left-wing}\right)\end{aligned}$$

then the partisanship score of a word is defined as,

$$partisanship(word) = \cos\left(\overrightarrow{word}, \frac{1}{6}\overrightarrow{rightleft}\right)$$

Notice that the division of *rightleft* by 6 is not necessary, as cosine similarity is scale-invariant.

[1] https://radimrehurek.com/gensim/models/word2vec.html

The partisanship scores were then z-scored separately for each model. Therefore, every word in an embedding model is assigned a partisanship score so that a lower score indicates that a word is more semantically similar to the political left, and a higher score that a word is more semantically similar to the political right. This approach was originally proposed by Kozlowski et al. (reference in the main text) who applied it to generate other semantic dimensions of social interest, such as affluence (rich vs. poor) and gender (feminine vs. masculine). Note also that prior work has shown that semantic dimensions can be constructed even with fewer words than those used here (12 words; 6 pairs) to construct the semantic dimension of partisanship (9).

To validate the semantic dimension of partisanship, we needed an externally provided vocabulary with annotated left-wing and right-wing words. To the best of our knowledge, such a vocabulary was not publicly available, and thus we developed one. We first extracted a list of 800 (non-unique) words from two popular pre-trained word embedding models, as follows. Using the model glove-twitter-200, we derived the 100 most similar words to the word *democrat*. We then did the same for the words *republican*, *democrats*, and *republicans*. We repeated this step using the model glove-wiki-gigaword-300. There were 327 unique words among these 800 words. We then recruited 7 undergraduate research assistants from our university to assess "*With which political party are the following words associated?*" with 3 options available for each word: *Democrats*, *Neither*, and *Republicans*. For 303 of the 327 words (93% of the words), at least 5 of the 7 research assistants provided the same assessment. Among these 303 words, 54 were assessed as being associated with Democrats and 57 with Republicans (for the rest 192 words, at least five research assistants chose *Neither*).

The following is the list of the 54 words associated with Democrats:
atheist, atheists, barack, biden, clinton, corzine, crist, cuomo, daschle, democrat, democratic, democrats, dems, dianne, dnc, dodd, feinstein, feminism, feminist, feminists, gephardt, gillibrand, gore, hillary, hipster, kennedy, kerry, ldp, left-wing, leftist, lib, liberal, liberalism, liberals, libs, lieberman, markey, massachusetts, mcgovern, murtha, naacp, ndp, obama, obamacare, pelosi, progressive, progressives, rangel, rodham, schumer, socialism, socialist, socialists, spd.

The following is the list of the 57 words associated with Republicans:
bjp, boehner, bush, catholic, catholics, centre-right, christian, christians, christie, conservador, conservatism, conservative, conservatives, cornyn, cpac, damato, dole, evangelical, evangelicals, fundamentalist, gingrich, giuliani, gop, gramm, hastert, huckabee, iowa, likud, lott, mccain, mcconnell, mitt, mormon, nationalist, newt, nra, palin, pataki, pro-life, reagan, repub, republican, republicans, repubs, right, right-wing, rightist, rnc, romney, santorum, tcot, teaparty, tories, tory, traditionalist, ukip, whig.

Then, for each of our models, we assessed the partisanship score of the words associated with Democrats and of the words associated with Republicans. The model based on the comments of left-wing users in left-wing subreddits (Figure 1a, main text) achieved d = 3.08. The model based on the comments of left-wing users in mixed-company subreddits (Figure 1b, main text) achieved d = 2.23. The model based on the comments of right-wing users in right-wing subreddits (Figure 1d, main text) achieved d = 3.14. The model based on the comments of right-wing users in mixed-company subreddits (Figure 1e, main text) achieved d = 2.08. Therefore, we observe that the distance between the words about Democrats and the words about Republicans was at least 2 SDs in each of the four models, suggesting successful validation of the dimension of partisanship in all these models. Similar conclusions were derived for the validation of partisanship in the four models in Figure 2 of the main text: for left-wing users in political left-wing subreddits, d = 2.85; for left-wing users in nonpolitical left-wing subreddits, d = 2.90; for right-wing users in political right-wing subreddits, d = 3.32; for right-wing users in nonpolitical right-wing subreddits, d = 2.64.

Constructing and validating the semantic dimension of morality. To construct the semantic dimension of morality, we devised a list of 18 pairs of words, with 3 pairs tapping general morality

and 3 pairs for each of the five moral foundations of the Moral Foundations Theory (care, fairness, loyalty, authority, sanctity):
[moral, immoral], [ethical, unethical], [righteous, wicked], [care, neglect], [peaceful, violent], [empathetic, apathetic], [fair, unfair], [unbiased, biased], [justice, injustice], [loyalty, treason], [fidelity, infidelity], [ally, enemy], [comply, defy], [respectful, disrespectful], [lawful, unlawful], [holy, unholy], [clean, filthy], [integrity, corruption]

The derivation of the morality scores for the words of each model was then similar to that for the dimension of partisanship.

We first validated the semantic dimension of morality for each model with the Moral Foundations Dictionary (10), which consists of words about virtues and vices. The model based on the comments of left-wing users in left-wing subreddits (Figure 1a, main text) achieved d = 2.20. The model based on the comments of left-wing users in mixed-company subreddits (Figure 1b, main text) achieved d = 2.32. The model based on the comments of right-wing users in right-wing subreddits (Figure 1d, main text) achieved d = 2.38. The model based on the comments of right-wing users in mixed-company subreddits (Figure 1e, main text) achieved d = 2.41. The models corresponding to Figure 2 achieved d between 2.09 and 2.34. These outcomes suggest successful validation of the semantic dimension of morality for all our 8 models.

To further validate the semantic dimension of morality, we also considered correlations with the extended Moral Foundations Dictionary (11). Each word in this dictionary is rated according to how relevant the word is to the five moral foundations, ranging from 0 (not relevant) to 1 (completely relevant). For example, the word “tortured” is rated as highly relevant to care, while the word “employed” is rated as irrelevant to care. We computed the dictionary-based (unsigned) morality score of a word as the mean across these five values, such that higher scores indicate the word refers more to moral foundations, in general. Notice that these ratings of moral relevance alone do not specify whether a word is about a virtue or a vice. Thus, we also used the ratings of all words on sentiment scores (which are part of this dictionary), ranging from −1 (most negative sentiment) to 1 (most positive sentiment). For example, the words “friendly” and “hostile” are both equally relevant to morality, but “friendly” is positive in sentiment and “hostile” is negative in sentiment. We assigned a negative sign to the morality score of a word if its sentiment scores were negative and a positive sign if its sentiment scores were positive, excluding words with mixed sentiment signs across different foundations. For all 8 models, the correlation between the model-based morality and the dictionary-based morality of the words was between .58 and .62. These adequately high correlations, which show little variation across our models, are therefore in line with our conclusion about the successful validation of the dimension of morality.

The dimension of valence. To provide further intrinsic evaluation for our word embedding models, we also constructed a semantic dimension of valence based on the 25 pleasant and the 25 unpleasant words used in Caliskan et al. (12), and then we examined the correlation of this dimension with the human judgments derived in Warriner et al. (13). For the four models corresponding to Figure 1 of the main text, this correlation was between .59 and .63, and for the four models corresponding to Figure 2 of the main text, this correlation was between .47 (left-wing users in political left-wing subreddits) and .62 (left-wing users in nonpolitical left-wing subreddits). Thus, the correlation between the dimension of valence in our models and the human judgments of valence was overall moderate-to-high, providing confidence in the trained embedding models.

Because morality is generally associated with valence (14), we examined whether the conclusions from the results in Figure 1 and Figure 2 are also derived when the impact of valence is taken into account. For each of the 8 models behind these two figures, we regressed

partisanship on both morality and valence to derive the partial correlation coefficient (henceforth denoted as r_p) between partisanship and morality.[2]

Beginning with the results that parallelize those in Figure 1, for left-wing users, we still observed a small difference in partisan moralization between partisan (r_p = −.20) and nonpartisan (r_p = −.18) subreddits. For right-wing users, there was a greater difference in their partisan moralization between partisan (r_p = .33) and nonpartisan subreddits (r_p = .18), although this difference was smaller than that reported in Figure 1 (compare to .40 versus .12). The partial correlation coefficient for right-wing users in nonpartisan subreddits was higher than the corresponding nonpartial correlation (.12 versus .18) because the partial correlation coefficient for valence was negative in that regression. From a statistical standpoint, this implies that controlling for morality, the direction of affective polarization reverses for right-wing users in nonpartisan subreddits—we find it difficult to believe this from a theoretical standpoint, and we rather believe it to be a statistical irregularity.

For the partial correlations corresponding to Figure 2, for left-wing users, we had r_p = .25 in political left-wing subreddits and r_p = .20 in nonpolitical left-wing subreddits. For right-wing users, we had r_p = .40 in political right-wing subreddits and r_p = .20 in nonpolitical right-wing subreddits.

Overall, we observe that partisanship and morality were correlated even after accounting for valence, although these correlations were generally smaller than those reported in the main text because of the correlation between morality and valence.

Avoiding overrepresentation of users. To avoid overrepresenting the highly active users, for users with more than 50 comments in a type of context (partisan or mixed-company), we randomly sampled 50 of their comments in that context type before developing the four word embedding models in Figure 1. The models corresponding to Figure 2 were developed based on data used for the models corresponding to Figure 1, as described below.

Paired bootstrap. Now, we describe the bootstrap method used to generate the results in Figures 1c and 1f of the main text. Notice that by construction of our dataset, all users had comments both in their ingroup partisan subreddits and in the mixed-company subreddits (between 10 and 50 comments in each type of subreddits). Thus, the models developed for these two types of subreddits are not independent, as individual users might have idiosyncrasies that are reflected in the semantics of the language in their comments, regardless of the context in which they are posted. To respect this dependency, in each bootstrap replication we first sampled users with replacement. That is, for the bootstrap results corresponding to Figure 1c, we sampled with replacement 671,979 (= $N_{\text{left-wing users}}$) users, and for the bootstrap results corresponding to Figure 1f, we sampled with replacement 430,148 (= $N_{\text{right-wing users}}$) users. Then, for a bootstrap replication corresponding to Figure 1c (1f), we formed a corpus in which the comments of a user in left-wing (right-wing) subreddits appeared as many times as that user appeared in the bootstrapped sample, and a corpus in which the comments of that user in mixed-company subreddits also appeared as many times as the user appeared in the bootstrapped sample. After the models were trained and the correlations between partisanship and morality were computed, the replication-specific difference between the correlations for partisan and mixed-company subreddits was taken. We performed 1,000 bootstrap replications, which implies that in Figures 1c and 1f combined, the total number of models trained was 4,000.

---

[2] For these analyses, the valence dimension was defined after excluding 10 of the 50 words used in Caliskan et al. that are explicitly about morality (honest, kill, honor, filth, peace, abuse, loyal, assault, caress, murder)—most of these words are literally part of the Moral Foundations Theory Dictionary. The validation results of this valence dimension defined on the subset of 40 words were virtually identical to those for the full set of 50 words.

The reported bootstrapped p-values are based on the proportions of the bootstrap replications against the null hypothesis. For instance, in Figure 1c, in 198 of the 1,000 replications (19.8%) the difference between the two correlations was negative. Notice that this proportion corresponds to a one-sided hypothesis test. To report a p-value for a two-sided hypothesis test, we doubled this proportion (39.6%), and thus in the main text we are reporting $P_{\text{bootstrap}} > .39$. Moreover, all the 1,000 bootstrapped correlations for the left-wing users in left-wing subreddits and all the 1,000 bootstrapped correlations for these users in mixed-company subreddits were negative. Thus, $P_{\text{bootstrap}} < .001$ is reported for the results corresponding to Figures 1a and 1b of the main text. In Figure 1f, all the 1,000 bootstrapped differences were positive, with all the 1,000 bootstrapped correlations for right-wing users in right-wing subreddits being positive. For such users in mixed-company subreddits, 23 of the 1,000 bootstrapped replications were negative, and thus we are reporting $P_{\text{bootstrap}} < .05$ for a two-sided hypothesis test.

Political and nonpolitical partisan subreddits. The four models corresponding to Figure 2 (main text) were developed by splitting the comments used for the models corresponding to Figures 1a and 1d (main text). The 291 left-wing subreddits consisted of 45 political and 246 nonpolitical subreddits, and the 176 right-wing subreddits consisted of 34 political and 142 nonpolitical subreddits.

The vast majority of all left-wing users (98%) and all right-wing users (91%) had comments in nonpolitical partisan subreddits. Thus, the models corresponding to Figure 1 (main text) and to nonpolitical partisan subreddits in Figure 2 (main text) were trained largely on the same users. However, only 18% of all left-wing users and 29% of all right-wing users had comments in political partisan subreddits. The reason our sample did not consist only of users with comments in all three kinds of subreddits (nonpartisan, political partisan, nonpolitical partisan) is that this would significantly reduce the number of users we examined since relatively few users had comments in political partisan subreddits. Notice, however, that the number of political partisan subreddits is quite smaller than the number of nonpolitical partisan subreddits, for both political sides.

To examine the possibility that the difference in our Figure 2 results for right-wing users (in political vs. nonpolitical right-wing subreddits) might be due to differences in the sets of users contributing to the two corresponding models in Figure 2, we considered only the right-wing users who had comments in both political and nonpolitical right-wing subreddits (20% of all right-wing users). We trained two models for these users, one based on their comments in political right-wing subreddits and one in nonpolitical right-wing subreddits. The results from these two models aligned with those presented in Figure 2, as the correlation between partisanship and morality was .36 in political right-wing subreddits and .21 in nonpolitical right-wing subreddits. Thus, even when we considered only the right-wing users who had comments in both political and nonpolitical right-wing subreddits, we observed a significant difference in partisan moralization across these two kinds of subreddits. This observation aligns with the conclusions derived from the results presented in Figure 2 of the main text (where not all users had comments in both political and nonpolitical partisan subreddits).

In Figure 2, the 95% confidence intervals were computed by multiplying bootstrapped standard errors (based on 1,000 replications) by ±1.96. Because not every user had comments in both political and nonpolitical partisan subreddits, we did not consider a paired bootstrap as in the results for Figure 1. However, we still sampled users with replacement, in each bootstrap replication. All the 4,000 bootstrapped correlations corresponding to Figure 2 (main text) were positive, and thus it holds that $P_{\text{bootstrap}} < .001$ for each group-context pair. In the comparison between left-wing and right-wing users in their respective political partisan subreddits, we compared 1,000 pairs of the (randomly paired) bootstrapped replications (15). The correlation of the left-wing users was higher than that of the right-wing users in only 1 of the 1,000 comparisons ($P_{\text{bootstrap}} < .002$). In similar comparisons of the right-wing users in political partisan subreddits against the left-wing users in nonpolitical partisan subreddits and the right-wing users in nonpolitical partisan subreddits, the correlation of the right-wing users in political partisan

subreddits was always higher ($P_{\text{bootstrap}} < .001$). To take into account the three simultaneous comparisons, in the main text we have reported $P_{\text{bootstrap-Bonferroni}} < .01$.

Other supporting information. The results of the four Pearson correlations between partisanship and morality depicted in Figure 1 of the main text were replicated when Spearman correlations were considered instead ($\rho = -.22$ for the results in Figure 1a, $\rho = -.23$ for the results in Figure 1b, $\rho = .38$ for the results in Figure 1d, and $\rho = .11$ for the results in Figure 1e), and when the correlations were taken only for the common words (N = 16,902 common words) of the four models ($r = -.22$ for the results in Figure 1a, $r = -.22$ for the results in Figure 1b, $r = .39$ for the results in Figure 1d, and $r = .12$ for the results in Figure 1e).

The mean (standard deviation) partisanship score across nonpartisan subreddits was −0.01 (0.14), across political left-wing subreddits −2.84 (0.72), across nonpolitical left-wing subreddits −2.47 (0.42), across political right-wing subreddits 2.92 (0.69), and across nonpolitical right-wing subreddits 2.38 (0.40).

To estimate the proportion of comments with political references, we used the LIWC dictionary (16), which includes a category about politics. We found that the proportion of comments with at least one word about politics was, for left-wing users: 3% in nonpartisan subreddits, 32% in political left-wing subreddits, and 4% in nonpolitical left-wing subreddits. For right-wing users, the corresponding proportions were 3% in nonpartisan subreddits, 24% in political right-wing subreddits, and 4% in nonpolitical right-wing subreddits. Thus, left-wing users made more political references in their political subreddits than right-wing users made in their political subreddits, but otherwise, the two sets of users were similar.

Moreover, we replicated the main text's Figure 1 and Figure 2 results using only comments from 2017-2022. Beginning with the results that parallelize those in Figure 1, for left-wing users, the correlation between partisanship and morality was $r = -.25$ in left-wing subreddits and $r = -.27$ in mixed-company subreddits. For right-wing users, this correlation was $r = .34$ in right-wing subreddits and $r = .13$ in mixed-company subreddits. For the results that parallelize those in Figure 2, for left-wing users, the correlation between partisanship and morality was $r = -.29$ in political left-wing subreddits and $r = -.28$ in nonpolitical left-wing subreddits. For right-wing users, this correlation was $r = .39$ in political right-wing subreddits and $r = .15$ in nonpolitical right-wing subreddits. Therefore, we observe that these correlations based only on comments from 2017-2022 (83% of all comments) were similar to those reported in the main text, where comments starting from 2006 were also included.

The 10 words in the left column of Table 1 (main text) are the 10 right-most words in Figure 1b (main text), excluding the 12 words about partisanship in the list presented above. Similarly, the 10 words in the right column of Table 1 (main text) are the 10 left-most words in Figure 1e (main text), excluding the same 12 words about partisanship.

The top-10 words that left-wing users associated with left-wing targets in mixed-company subreddits were: vinci, humanities, café, uc, revolution, oxford, nobel, karl, mona, and berkeley.

The top-10 words that right-wing users associated with right-wing targets in mixed-company subreddits were: allocation, bonds, distributions, stocks, shares, implications, td, equities, implication, and estimates.

The top-10 words that left-wing users associated with right-wing targets in left-wing subreddits were: evangelical, bigoted, anti-lgbt, fundie, evangelicals, homophobes, religious, bigots, anti-gay, and homophobic.

The top-10 words that right-wing users associated with left-wing targets in right-wing subreddits were: infested, ridden, retardation, sjw, libtard, francisco, cesspool, shithole, inducing, and diarrhea.

The top-10 words that left-wing users associated with left-wing targets in left-wing subreddits were: dsa, trotsky, comrades, rosa, revolutions, vanguard, reformist, luxemburg, leninism, and socdem.

The top-10 words that right-wing users associated with right-wing targets in right-wing subreddits were: ron, titans, principled, bernier, rand, 2024, mcconnell, values, mtg, and rb.

Moderators and usernames including the term “bot” were excluded from our sets of users.

Reddit comments were extracted from the Pushshift dataset (17).

SI References

1. Gerz, D., Vulić, I., Hill, F., Reichart, R., & Korhonen, A. (2016). SimVerb-3500: A Large-Scale Evaluation Set of Verb Similarity. In *Proceedings of the 2016 Conference on Empirical Methods in Natural Language Processing*. Association for Computational Linguistics.
2. Finkelstein, L., Gabrilovich, E., Matias, Y., Rivlin, E., Solan, Z., Wolfman, G., & Ruppin, E. (2001, April). Placing search in context: The concept revisited. In *Proceedings of the 10th International Conference on World Wide Web* (pp. 406-414).
3. Luong, M. T., Socher, R., & Manning, C. D. (2013, August). Better word representations with recursive neural networks for morphology. In *Proceedings of the 17th Conference on Computational Natural Language Learning* (pp. 104-113).
4. Hill, F., Reichart, R., & Korhonen, A. (2015). Simlex-999: Evaluating semantic models with (genuine) similarity estimation. *Computational Linguistics*, *41*(4), 665-695.
5. Bruni, E., Tran, N. K., & Baroni, M. (2014). Multimodal distributional semantics. *Journal of Artificial Intelligence Research*, *49*, 1-47.
6. Radinsky, K., Agichtein, E., Gabrilovich, E., & Markovitch, S. (2011, March). A word at a time: computing word relatedness using temporal semantic analysis. In *Proceedings of the 20th International Conference on World Wide Web* (pp. 337-346).
7. Halawi, G., Dror, G., Gabrilovich, E., & Koren, Y. (2012, August). Large-scale learning of word relatedness with constraints. In *Proceedings of the 18th ACM SIGKDD International Conference on Knowledge Discovery and Data Mining* (pp. 1406-1414).
8. Caselles-Dupré, H., Lesaint, F., & Royo-Letelier, J. (2018, September). Word2vec applied to recommendation: Hyperparameters matter. In *Proceedings of the 12th ACM Conference on Recommender Systems* (pp. 352-356).
9. Charlesworth, T. E., Caliskan, A., & Banaji, M. R. (2022). Historical representations of social groups across 200 years of word embeddings from Google Books. *Proceedings of the National Academy of Sciences*, *119*(28), e2121798119.
10. Graham, J., Haidt, J., & Nosek, B. A. (2009). Liberals and conservatives rely on different sets of moral foundations. *Journal of Personality and Social Psychology*, *96*(5), 1029.
11. Hopp, F. R., Fisher, J. T., Cornell, D., Huskey, R., & Weber, R. (2021). The extended Moral Foundations Dictionary (eMFD): Development and applications of a crowd-sourced approach to extracting moral intuitions from text. *Behavior research methods*, *53*, 232-246.
12. Caliskan, A., Bryson, J. J., & Narayanan, A. (2017). Semantics derived automatically from language corpora contain human-like biases. *Science*, *356*(6334), 183-186.
13. Warriner, A. B., Kuperman, V., & Brysbaert, M. (2013). Norms of valence, arousal, and dominance for 13,915 English lemmas. *Behavior Research Methods*, *45*, 1191-1207.
14. Decety, J., & Howard, L. H. (2013). The role of affect in the neurodevelopment of morality. *Child Development Perspectives*, *7*(1), 49-54.
15. Rousselet, G. A., Pernet, C. R., & Wilcox, R. R. (2021). The percentile bootstrap: a primer with step-by-step instructions in R. *Advances in Methods and Practices in Psychological Science*, *4*(1), 2515245920911881.

16. Boyd, R. L., Ashokkumar, A., Seraj, S., & Pennebaker, J. W. (2022). The development and psychometric properties of LIWC-22. *Austin, TX: University of Texas at Austin*, *10*.
17. Baumgartner, J., Zannettou, S., Keegan, B., Squire, M., & Blackburn, J. (2020, May). The pushshift reddit dataset. In *Proceedings of the international AAAI conference on web and social media* (Vol. 14, pp. 830-839).
